# Parental Knowledge, Attitude, and Practice on Tobacco Use, Smoking Cessation, and Children's Environmental Tobacco Smoke Exposure

**DOI:** 10.3389/fpubh.2021.733667

**Published:** 2021-11-24

**Authors:** Siyu Dai, Chun Ting Au, Michael Ho Ming Chan, Richard Kin Ting Kam, Albert Martin Li, Kate Ching-Ching Chan

**Affiliations:** ^1^Department of Pediatrics, Faculty of Medicine, The Chinese University of Hong Kong, Hong Kong, China; ^2^School of Clinical Medicine, Hangzhou Normal University, Hangzhou, China; ^3^Department of Chemical Pathology, Prince of Wales Hospital, Hong Kong, China

**Keywords:** child, environmental tobacco smoke, KAP, parent, smoke-free policy, smoking cessation, tobacco use

## Abstract

**Background:** Environmental tobacco smoke (ETS) exposure in children ranks one of the major public health problems in our time. Poor parental knowledge, attitude, and practice (KAP) on ETS often contribute to worse exposure of the kids. Thus, we aimed to document parental KAP regarding tobacco use, smoking cessation and children's ETS exposure, and to analyse how knowledge and attitude relate to practice.

**Methods:** Self-administered KAP questionnaires were distributed to smoking parents recruited from the pediatric unit at the Prince of Wales Hospital, which provides pediatric service to a population of 1.2 million in Hong Kong. The 60-item questionnaire had a range of 0–38 for knowledge, 0–44 for attitude, and 0–40 for practice. Descriptive analyses were performed for KAP response, regression analyses were performed for the exploration of associations and identification of predictive indicators.

**Results:** 145 smoking parents (mean age: 38.0 ± 6.7 yrs.; male: 85.5%) were included. Less than half (39.3%) of them reported a smoke-free policy at home. Among those parents who had private cars, less than half (45.2%) of them had smoke-free policy in their car that they never smoked in the car. Only 25.5% of the participants correctly answered ≥70% of the knowledge questions, and 11.8 % of the participants gave favorable responses to ≥70% of the attitude questions. The total knowledge and the total attitudes score were positively associated (*r* = 0.49, 95% CI: 0.35–0.79, *p* < 0.001), yet they were only modestly correlated with parental practice on children's ETS exposure. By multivariate regressions, potential predictive factors for more favorable parental KAP included higher household income, lower parental nicotine dependence level and breastfeeding practice.

**Conclusions:** Parental KAP related to tobacco use and children's ETS exposure needs improvement to address the significant gap between recommended and actual practice. The weak association between knowledge and practice suggested that parental education alone is not adequate to combat ETS exposure in children.

## Introduction

Parental smoking is the main source of environmental tobacco smoke (ETS) exposure in children. Continued efforts are raised to emphasize the importance of parental smoking cessation and smoking behavioral modifications to protect children from ETS exposure (1, 2). There is no safe exposure level of ETS, smoking cessation is the best strategy to prevent children from the hazardous exposure. However, tobacco is highly addictive and nicotine dependence is not easy to resolve (3). Previous research found that majority of the smoking parents are unwilling to quit (4, 5). Therefore, parents are recommended to at least reduce their tobacco consumption and initiate smoke-free policy at home and in private vehicles if they are not ready to quit (6). Noteworthily, even without second-hand smoke (SHS), smokers and environment can carry toxic residues causing exposure to people in contact subsequently which is known as thirdhand smoke (THS) (7, 8). Previous studies found that nicotine and nicotine related alkaloids can be removed from cotton fabrics by washing, and suggested washing might be a simple remediation procedure for THS reduction (9, 10).

Globally, information about parental practice related to children's ETS exposure is scarce. Existing literature has only explored the practice of private smoke-free policy among parents. The updated establishment rate of home smoke-free policy was reported to be 79.5% in the US, 76.0% in Poland, and 14.3% in Guangxi province of China, while the rate of car smoke-free policy was 81.8% in the US and 80% in Canada (11–14). Nonetheless, the actual practice in Hong Kong has not been examined (15). The level of knowledge, attitude, and practice (KAP) in individuals was closely linked to the efficiency of illness management, the response toward medical treatment as well as the results of health promotion (16–18). Furthermore, health behavior could be associated with one's health knowledge and attitude, although better knowledge and attitude might not promise a better practice (19, 20). Exploration of smoking parents' perceived susceptibility and severity of children's ETS exposure, benefits and barriers of smoking cessation will provide us information to improve the interventions for cessation in parents and reduction of ETS exposure in children (20–22).

The majority of the existing smoking related KAP studies were targeted at active smoking behavior only, and their target populations were general smokers or health care workers (23–25). Winickoff et al. found the widespread unawareness of ETS-related hazards especially THS among smoking parents in the US (26). Hasniah et al. found that poor parental awareness of ETS hazards contributed to more severe exposure in children (27). In Hong Kong, a previous study carried out two decades ago reported that many mothers of sick children were unaware of the ETS hazards, and their actions toward their children's exposure were inadequate (28). However, fathers were very often the main source of children's ETS and their knowledge and attitude were not examined (28). According to our best knowledge, there is no KAP study regarding children's passive smoke exposure yet.

Understanding of smoking parents' KAP can be the cornerstone of a successful intervention in order to reduce ETS exposure in children. Thus, we designed this study to evaluate the KAP of smoking parents regarding tobacco use, smoking cessation, and children's ETS exposure in Hong Kong. Our secondary objectives were to explore whether better knowledge and attitude were related to more favorable parental practice and to identify potential predictors for favorable KAP.

## Methods

This KAP survey study was a sister project of our smoking reduction randomized controlled trial (RCT) (clinicaltrials.gov: NCT03879889) for parents of pediatric patients. Written informed consent was obtained from the smoking parent. At the baseline data collection before the randomization for the RCT, the participants were invited to complete a standardized questionnaire and the KAP questionnaire. The standardized questionnaire included collection of information on parental [including parental nicotine dependence level assessed by Fagerstrom Test for Nicotine Dependence (FTND)] and child's demographics and clinical characteristics. The smoking parents and the children were also invited to provide their urine samples in the same visit for the validation of their tobacco exposure level.

### Ethics Approval

This study was conducted in accordance with the Declaration of Helsinki and was approved by the Joint Chinese University of Hong Kong-New Territories East Cluster Research Ethics Committee (CRE 2016.024-T).

### Participants

Smoking parents were recruited from the outpatient and inpatients pediatric units of the Prince of Wales Hospital (PWH) by convenience sampling method. PWH is the teaching hospital of the Chinese University of Hong Kong, and it is a major tertiary hospital which provides pediatric service to a population of 1.2 million in Hong Kong. This KAP study shared the same inclusion and exclusion criteria with the RCT. Inclusion criteria were smoking parents living with children aged <18 years who attended our clinics or wards; exclusion criteria were families with children in foster care or with unclear custody and smoking pediatric patients. Based on a previous study performed in Guangxi, China, the prevalence of home smoke-free policy was 14.3% (14). For this estimated prevalence, a sample size of 145 provided a confidence level of 95% with 5.7% margin of error. The actual prevalence of home smoke-free policy identified in this KAP study was 39.3%, thus the sample size of 145 provided a confidence level of 95% with a 7.9% margin of error.

### Development and Validation of the KAP Scale

We designed the KAP scale by referring to the WHO KAP development guideline (29), several KAP scale development studies (30, 31), previous smoking related KAP studies (23–25), and the Global Adult Tobacco Survey. Health belief model (HBM) was employed in the scale development. The HBM views health behavioral change as based on a rational appraisal of the balance between the barriers to and benefits of action.

After panel revision and pilot study, the finalized scale had an overall Cronbach's alpha coefficient of 0.97. The Cronbach's alpha coefficients for the knowledge, attitude, and practice sections were 0.92, 0.98, and 0.77, respectively. Each item was marked with one score for every favorable response and no score for unfavorable or unknown response, except for the items in parental practice section regarding children's ETS exposure, in which 5-point Likert scale was adopted (options “often” or “always” were regarded as favorable responses). For the practice section, the constructed index included parental implementation of smoke-free policy at home and in private car, which are the most important policies to protect children from ETS exposure, and also practices regarding children's SHS-specific (opening a door or window when smoking, switching on ventilation during smoking and keeping distance away from children when smoking) and THS-specific (washing practices including rinsing mouth, taking a shower, washing hand, and changing clothes) exposure. The scale had a score range of 0–38 for total knowledge (TK), 0–44 for total attitude (TA), and 0–40 for parental practice regarding children's ETS exposure (TP-ETS). Higher scores indicated more favorable KAP. Trained research personnel supervised the questionnaire completion, face-to-face in-depth interviews were performed with unclear points clarified. The missing data rate was very low (<5% for all items).

### Data Analysis

Descriptive analyses were performed for the demographic characteristics and documentation of the KAP. The total scores of each section and score of each sub-section of KAP were described as mean with standard deviation (SD) if normally distributed or median with interquartile range (IQR) if non-normally distributed. The proportions of participants who had favorable responses for ≥ 70% items of parental practice regarding children's ETS exposure, ≥70% items of the knowledge section, and ≥ 70% items of the attitude section were calculated. The relationships between knowledge, attitude, and practice, and the identification of the predictors were assessed by linear and logistic regressions as appropriate (23, 24, 27). We used statistical software packages SPSS (version 23.0 for Windows; SPSS Inc.) for all the analyses. *P* < 0.05 was regarded as significant.

## Results

**Figure 1:**
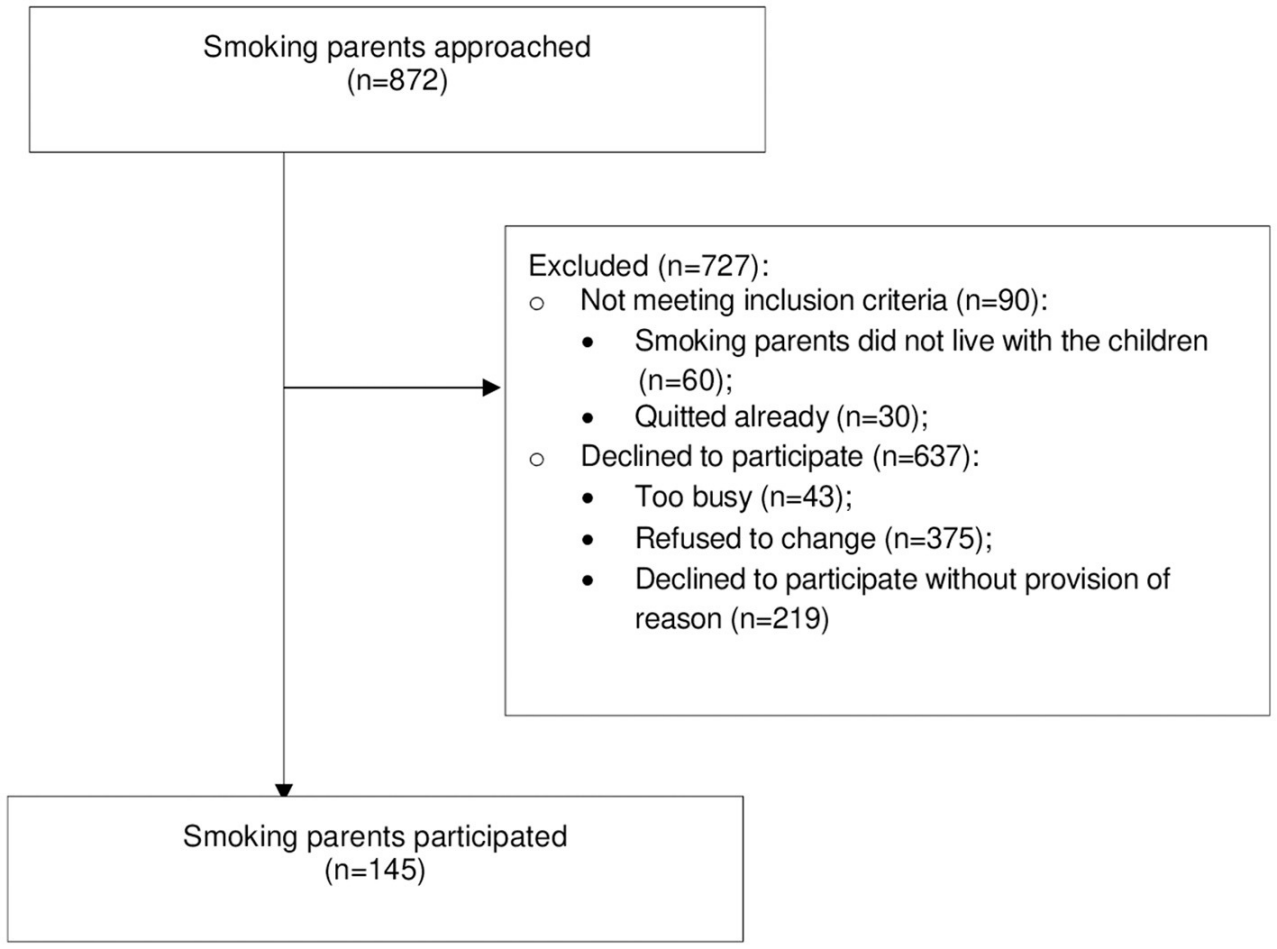
Flow diagram of study participants.

During October 2017 to July 2019, a total of 872 eligible smoking parents (self-reported current smokers meeting our inclusion criteria) were approached, and a total of 145 parents participated in this KAP study, resulting in a 16.6% response rate (Figure 1).

Demographic and smoking history of the parents are shown in Table 1. Most of them were mid-aged fathers and more than half of them had an overcrowded household living area. 29.0% of the participating parents had current or previous chronic medical conditions. For smoking conditions, most of our participants were daily smokers with moderate nicotine dependence level (FTND score: 3.8 ± 2.1). Their mean tobacco consumption was 15.4 ± 7.4 cigarettes/day, and their median urine cotinine concentration was 959.8 (542.8–1452.2) μg/L. Characteristics of the children are shown in Table 2. Median age of the children was 2.7 (1.2–6.3) years. Most of the kids had chronic medical conditions (90.9%), and 35.0% of them had chronic respiratory tract disease. The median urine cotinine concentration of the children was 0.34 (0.0–1.18) μg/L.

**Table 1:** Demographic characteristics and smoking history of the smoking parents (*N* = 145).

**Characteristics**	***N*** **= 145**
Age (years)	38.0 ± 6.7
Male sex, *n* (%)	124 (85.5%)
Education level: Primary school or below, *n* (%)	5 (3.5%)
Secondary school, *n* (%)	109 (75.7%)
Tertiary education or above, *n* (%)	30 (20.8%)
Monthly household income ≤ HKD20,000, *n* (%)	50 (34.7%)
Overcrowding of living place^∧^, *n* (%)	79 (54.9%)
Current or previous chronic medical conditions, *n* (%)	42 (29.0%)
Daily smoker, *n* (%)	130 (89.6%)
Average daily smoking consumption in the past 1 month (cigarettes/day):	15.4 ± 7.4
Urine cotinine concentration (μg/L)^*^, median (IQR)	959.8 (542.8–1452.2)
FTND score^#^, mean ± SD	3.8 ± 2.1
Previous quit attempt	96 (66.2%)
Motivation stage^&^: Pre-contemplation, *n* (%)	43 (29.7%)
Contemplation, *n* (%)	38 (26.2%)
Preparation, *n* (%)	64 (44.1%)
Smoking spouse, *n* (%)	24 (16.6%)

∧ *Overcrowding of living place was defined as a living space of ≤ 5.5 m^2^/person in accordance with the guideline of the Hong Kong Housing Authority.*

# *FTND score: 1–2 points indicates low dependence; 3–4 points indicates low to moderate dependence; 5–7 points indicates moderate dependence; >8 points indicates high dependence.*

& *Pre-contemplation: not intending to quit smoking in the next 6 months; Contemplation: intending to quit in the next 6 months but not in the next 30 days; Preparation: intending to quit in the next 30 days.*

* *Urine samples were available for 128 parents*.

**Table 2:** Demographic and clinical characteristics of the pediatric patients (*N* = 143).

**Characteristics**	***N*** **= 143**
Age (years), median (IQR)	2.7 (1.2–6.3)
Male sex, *n* (%)	83 (55.6%)
Existing chronic medical conditions^#^, *n* (%)	130 (90.9%)
Existing chronic respiratory tract diseases^∧^, *n* (%)	50 (35.0%)
Current or previous allergic rhinitis, *n* (%)	36 (25.2%)
Current or previous asthma, *n* (%)	15 (10.5%)
Current or previous eczema, *n* (%)	26 (18.2%)
Current or previous chronic lung disease, *n* (%)	5 (3.5%)
Parental perception on child's health status (Scale of 1–5)^@^, mean ± SD	3.5 ± 0.9
Need of long-term medication, *n* (%)	20 (14.0%)
Premature at birth (<37 weeks' gestation), *n* (%)	24 (16.8%)
Presence of other household smoker(s), *n* (%)	20 (14.0%)
Urine cotinine concentration (μg/L)^*^, median (IQR)	0.34 (0.0–1.18)

# *Including chronic respiratory tract diseases, heart disease, developmental problems, allergic rhinitis, eczema, asthma.*

∧ *Including allergic rhinitis, asthma, chronic lung diseases.*

@ *A Likert scale of 1 to 5; 1-very poor health condition, 5-very good health condition.*

* *Valid baseline urine samples were available for 89 children*.

Descriptive analysis results of parental practice are shown in Table 3. Only half of the parents stated that they were intending to quit in a short time (in 30 days: 44.1%; in 6 month: 64.8%). As the most important strategies to prevent children from ETS exposure, 39.3% of the participants stated that they had smoke-free policy at home, and among the 42 parents who had private cars, 45.2% of them stated that they had smoke-free policy in the car that they never smoked in their cars. Most of the parents stated they “often” or “always” opened the door/window when smoking at home (93.8%) and meant to keep distance from the children when smoking (91.0%), while much fewer of them would switch on the ventilation when smoking at home (29.7%). As for THS exposure, quite few of the smoking parents would “often” or “always” carry out “washing practice” including “mouth rinsing” (30.3%), “shower” (14.5%), “hand washing” (58.6%), and “changing clothes” (17.3%) after smoking and before body contact with the children. Very few of the parents had used local smoking cessation services including cessation hotline (11.0%) and cessation clinics (6.9%) previously. Only 14.5% of the parents had received nicotine replacement therapy previously, and none of them had received bupropion nor valenkelin for cessation treatment.

**Table 3:** Parental practice on tobacco use and smoking cessation (*N* = 145).

	***N*** (%)					
Intending to quit in the next 30 days	64 (44.1%)					
Intending to quit in the next 6 months	94 (64.8%)					
Have smoking ban policy						
At home	57 (39.3%)					
In private car (if applicable)^*^	19 (45.2%)					
Smoking cessation service type used previously:						
Smoking cessation hotline	16 (11.0%)					
Smoking cessation clinics	10 (6.9%)					
Smoking cessation medication used previously:						
Nicotine replacement therapy	21 (14.5%)					
Bupropion or Valenkelin	0 (0.0%)					
	**Never** ***n*** **(%)**	**Rarely** ***n*** **(%)**	**Sometimes** ***n*** **(%)**	**Often** ***n*** **(%)**	**Always** ***n*** **(%)**	**Not applicable** ***n*** **(%)**
Open the door/window when smoking at home	6 (4.1%)	2 (0.7%)	2 (0.7%)	12 (8.3%)	125 (85.5%)	1 (0.7%)
Switch on the ventilation when smoking at home	12 (8.3%)	28 (19.3%)	35 (24.1%)	30 (20.7%)	13 (9.0%)	27 (18.6)
Keep distance from the children when smoking	8 (5.5%)	1 (0.7%)	4 (2.8%)	15 (10.3%)	117 (80.7%)	0 (0.0%)
Keep 3 m or above distance from the children when smoking	8 (5.5%)	4 (2.8%)	11 (7.6%)	15 (10.3%)	107 (73.8%)	0 (0.0%)
Open the window when smoking in private car^*^	8 (19.0%)	1 (2.4%)	2 (4.8%)	4 (9.5%)	25 (59.5%)	2 (4.8%)
Rinse the mouth, after smoking and before body contact with the children	25 (17.2%)	28 (19.3%)	41 (28.3%)	17 (11.7%)	27 (18.6%)	7 (4.8%)
Take a shower, after smoking and before body contact with the children	40 (27.6%)	41 (28.3%)	34 (23.4%)	13 (9.0%)	8 (5.5%)	9 (6.2%)
Wash hands, after smoking and before body contact with the children	15 (10.3%)	9 (6.2%)	30 (20.7%)	26 (17.9%)	59 (40.7%)	6 (4.1%)
Change clothes, after smoking and before body contact with the children	40 (27.6%)	35 (24.1%)	37 (25.5%)	11 (7.6%)	14 (9.7%)	8 (5.5%)

* *42 out of the 145 smoking parents reported that they had private cars*.

Documentation of parental KAP scores is shown in Table 4. The mean of total practice scores regarding children's ETS exposure was 22.6 ± 6.0, and only 17.9% of the participants gave favorable response to 70% or more of the items. Mean total knowledge scores was 21.3 ± 8.0, and 25.5% of the participants gave correct answer to 70% or more of the items. The mean of total attitude scores was 20.9 ± 8.5, and 11.8% of the participants gave positive response to 70% or more of the items. Most of the parents (79.3%) had relatively favorable practice regarding children's SHS exposure, while their practice for THS was poor. Most of the parents had relatively good knowledge for K1 (definitions of active smoke, SHS, THS and E-cig), K5 (knew that their smoking behavior mattered), and K6 (knew that better ventilation could help reducing the exposure). However, parental knowledge for K2 (general harm of tobacco exposure) and K3 (specific harm on infants and children) were insufficient. In the attitude section, most of the parents had positive attitude on A3 (agreed that tobacco exposure attributed harm was serious) and A4 (agreed there could be many potential “positive” changes brought by smoking cessation). However, surprisingly low scores were obtained for A5 (parents were afraid of the changes brought by smoking cessation such as nicotine withdrawal symptom and the potential side effect of the cessation medications), A6 (participants did not believe their families and friends could help with their cessation), and A7 (participants lacked confidence in quitting by themselves). The baseline knowledge and attitude total scores were positively associated with each other (*r* = 0.49, 95% CI: 0.35–0.79, *p* < 0.001), while neither of them was associated with private smoke-free policy.

**Table 4:** Descriptive analyses of parental KAP scores and association with smoke ban policy (*N* = 145).

	**Mean ± SD**	**Percentage of participants answered 70% or more of the items favorably**	***r*** **(with smoke ban policy at home)**	***P*** **-value**
Total Practice scores regarding children's ETS exposure^@^ (TP-ETS) Full mark is 40	22.6 ± 6.0	17.9%	0.18 (0.15, 4.16)	**0.04**
Practice scores regarding children's SHS exposure^*^ Full mark is 20	14.4 ± 3.3	79.3%	−0.02 (−1.27, 0.95)	0.78
Practice scores regarding children's THS exposure^&^ Full mark is 20	8.1 ± 4.9	11.0%	0.23 (0.72, 3.91)	**0.005**
Total Knowledge scores (TK) Full mark is 38	21.3 ± 8.0	25.5%	−0.72 (−3.75, 1.75)	0.47
Basic concepts (K1) Full mark is 4	3.0 ± 1.1	78.1%	−0.03 (−0.40, 0.38)	0.97
General harmfulness of tobacco exposure (K2) Full mark is 6	3.3 ± 2.1	46.0%	0.11 (−0.26, 1.17)	0.21
Specific tobacco harmfulness on infants and children (K3) Full mark is 6	4.0 ± 3.0	50.4%	0.04 (−0.78, 1.27)	0.64
Harmful substances of cigarette (K4) Full mark is 6	3.2 ± 1.9	26.5%	0.03 (−0.54, 0.76)	0.74
Whether my smoking behavior matter (K5) Full mark is 4	2.7 ± 1.5	64.4%	−0.18 (−1.12, 0.05)	0.08
Methods to reduce tobacco exposure level (K6) Full mark is 6	4.6 ± 2.2	75.9%	−0.03 (−0.91, 0.64)	0.69
Smoking cessation services and medication (K7) Full mark is 6	1.7 ± 1.8	18.2%	−0.10 (−0.95, 0.28)	0.29
Total Attitude scores (TA) Full mark is 44	20.9 ± 8.5	11.8%	−0.05 (−3.76, 2.01)	0.55
Attitude on my smoking behavior (A1) Full mark is 11	3.2 ± 2.4	4.4%	−0.002 (−0.83, 0.82)	0.99
Attitude on tobacco control policy (A2) Full mark is 4	2.0 ± 1.6	46.0%	0.11 (−0.19, 0.90)	0.20
Attitude on tobacco attributed health harm (A3) Full mark is 3	2.3 ± 1.1	80.3%	−0.10 (−0.62, 0.16)	0.25
Attitude on potential “positive” changes brought by smoking cessation (A4) Full mark is 4	2.9 ± 1.3	75.9%	−0.04 (−0.56, 0.35)	0.65
Attitude on potential “negative” changes brought by smoking cessation (A5) Full mark is 6	1.1 ± 1.6	8.8%	0.08 (−0.29, 0.78)	0.36
Attitude on taking actions (smoking cessation/reduction) (A6) Full mark is 6	1.7 ± 1.6	19.1%	0.007 (−0.55, 0.59)	0.94
Attitude on smoking cessation/reduction by oneself (A7) Full mark is 10	3.0 ± 2.0	7.1%	0.07 (−0.63, 1.15)	0.57

@ *8 items (For each item, a Likert scale of 1–5; 1-never, 5-always) were included: (1) How often do you keep distance from your children when smoking at home? (2) How often do you keep 3 m or above distance from your children when smoking at home? (3) How often do you open the door/window when smoking at home? (4) How often do you switch on the ventilation when smoking at home? (5) After smoking, how often do you rinse the mouth before body contact with your children? (6) After smoking, how often do you take a shower before body contact with your children? (7) After smoking, how often do you wash your hand before body contact with your children? (8) After smoking, how often do you change your cloth before body contact with your children?*

* *Four items (For each item, a Likert scale of 1–5; 1-never, 5-always) were included: (1) How often do you keep distance from your children when smoking at home? (2) How often do you keep 3 m or above distance from your children when smoking at home? (3) How often do you open the door/window when smoking at home? (4) How often do you switch on the ventilation when smoking at home?*

& *Four items (For each item, a Likert scale of 1–5; 1-never, 5-always) were included: (1) After smoking, how often do you rinse the mouth before body contact with your children? (2) After smoking, how often do you take a shower before body contact with your children? (3) After smoking, how often do you wash your hand before body contact with your children? (4) After smoking, how often do you change your cloth before body contact with your children?*

*Bold value stands for p < 0.05*.

The associations between parental knowledge, attitude and practice regarding children's ETS (the total ETS exposure, SHS-specific exposure only and THS-specific exposure only) are shown in Table 5. Smoking parents with higher total knowledge scores (*r* = 0.19, 95% CI: 0.02–0.27, *p* = 0.03) and higher total attitude scores (*r* = 0.19, 95% CI: 0.02–0.25, *p* = 0.02) appeared to have better practice on children's ETS except for private smoke-free policy. The total knowledge scores (*r* = 0.19, 95% CI: 0.009–0.15, *p* = 0.03) and total attitude scores (*r* = 0.19, 95% CI: 0.01–0.14, *p* = 0.02) were also positively associated with parental scores of practices regarding children's SHS-specific exposure.

**Table 5:** Associations between parental total Knowledge, total Attitude, and Practice regarding children's ETS exposure (*N* = 145).

	***r*** **(with parental practice regarding children's ETS exposure)^@^**	***P*** **-value**	***r*** **(with parental practice regarding children's SHS exposure)^#^**	***P*** **-value**	***r*** (with parental practice regarding children's THS exposure)^*^	***P*** **-value**
Total Knowledge scores (TK)	0.19 (0.02, 0.27)	**0.03**	0.19 (0.009, 0.15)	**0.03**	0.10 (−0.04, 0.16)	0.22
Basic concepts (K1)	0.03 (−0.76, 1.04)	0.75	0.10 (−0.20, 0.78)	0.24	−0.03 (−0.89, 0.59)	0.69
General harmfulness of tobacco exposure (K2)	0.20 (0.10, 1.06)	**0.02**	0.23 (0.10, 0.62)	**0.006**	0.09 (−0.19, 0.62)	0.30
Specific tobacco harmfulness on infants and children (K3)	0.12 (−0.09, 0.59)	0.15	0.19 (0.03, 0.40)	**0.02**	0.02 (−0.25, 0.32)	0.81
Harmful substances of cigarette (K4)	0.23 (0.20, 1.25)	**0.007**	0.18 (0.03, 0.61)	**0.03**	0.16 (−0.03, 0.85)	0.07
Whether my smoking behavior matter (K5)	0.07 (−0.50, 1.05)	0.70	0.08 (−0.26, 0.66)	0.40	0.02 (−0.56, 0.71)	0.81
Methods to reduce tobacco exposure level (K6)	0.14 (−0.09, 0.83)	0.12	0.08 (−0.13, 0.37)	0.35	0.11 (−0.13, 0.63)	0.20
Smoking cessation services and medication (K7)	−0.002 (−0.58, 0.56)	0.98	0.04 (−0.23, 0.39)	0.62	−0.03 (−0.56, 0.39)	0.72
Total Attitude scores (TA)	0.19 (0.02, 0.25)	**0.02**	0.19 (0.01, 0.14)	**0.02**	0.10 (−0.04, 0.15)	0.21
Attitude on my smoking behavior n(A1)	−0.004 (−0.43, 0.42)	0.97	−0.002 (−0.24, 0.23)	0.98	−0.003 (−0.36, 0.35)	0.97
Attitude on tobacco control policy (A2)	0.21 (0.17, 1.43)	**0.01**	0.25 (0.18, 0.86)	**0.003**	0.09 (−0.25, 0.81)	0.29
Attitude on tobacco attributed health harm (A3)	0.04 (−0.62, 0.94)	0.68	0.16 (−0.03, 0.94)	0.06	0.03 (−0.60, 0.88)	0.71
Attitude on potential “positive” changes brought by smoking cessation (A4)	−0.04 (−0.56, 0.35)	0.65	0.09 (−0.20, 0.65)	0.29	−0.02 (−0.71, 0.58)	0.84
Attitude on potential “negative” changes brought by smoking cessation (A5)	0.16 (−0.05, 1.23)	0.07	−0.03 (−0.41, 0.30)	0.77	0.20 (0.12, 1.17)	**0.02**
Attitude on taking actions (smoking cessation/reduction) (A6)	0.72 (−0.40, 0.85)	0.48	−0.04 (−0.43, 0.25)	0.62	0.10 (−0.20, 0.82)	0.23
Attitude on smoking cessation/reduction by oneself (A7)	0.008 (−0.62, 0.67)	0.94	−0.19 (−0.68, 0.04)	0.08	0.15 (−0.17, 0.86)	0.19

@ *8 items (For each item, a Likert scale of 1–5; 1-never, 5-always) were included: (1) How often do you keep distance from your children when smoking at home? (2) How often do you keep 3 m or above distance from your children when smoking at home? (3) How often do you open the door/window when smoking at home? (4) How often do you switch on the ventilation when smoking at home? (5) After smoking, how often do you rinse the mouth before body contact with your children? (6) After smoking, how often do you take a shower before body contact with your children? (7) After smoking, how often do you wash your hand before body contact with your children? (8) After smoking, how often do you change your clothes before body contact with your children?*

# *Four items (For each item, a Likert scale of 1–5; 1-never, 5-always) were included: (1) How often do you keep distance from your children when smoking at home? (2) How often do you keep 3 m or above distance from your children when smoking at home? (3) How often do you open the door/window when smoking at home? (4) How often do you switch on the ventilation when smoking at home?*

* *Four items (For each item, a Likert scale of 1–5; 1-never, 5-always) were included: (1) After smoking, how often do you rinse the mouth before body contact with your children? (2) After smoking, how often do you take a shower before body contact with your children? (3) After smoking, how often do you wash your hand before body contact with your children? (4) After smoking, how often do you change your clothes before body contact with your children?*

*Bold value stands for p < 0.05*.

As for predictors for more favorable KAP, the results from the univariate and multivariate analyses are shown in Supplementary Tables 1, 2. According to the multivariate logistic regression and linear regression analyses, factors associated with more favorable KAP were: higher monthly household income, lower parental nicotine dependence level, and children who had been breastfed.

## Discussion

In this KAP study of smoking parents of pediatric patients, a wide gap was observed between the actual and the recommended practice. The majority of the parents demonstrated insufficient knowledge and unfavorable attitude on tobacco use, smoking cessation and children's ETS exposure, which might partially explain the unfavorable behaviors. Parental knowledge and attitude were modestly associated with some favorable parental practice, nonetheless, they were not associated with household smoke-free policy which is vital to prevent children from the harmful ETS exposure.

Therefore, education alone may not be sufficient enough to change parents' behaviors and additional strategies, such as cessation program with pharmacological support, might be important components of an effective ETS reduction intervention (32, 33). Combined efforts of public smoke-free policy, household smoking ban, strong support for smoking cessation as well as ETS related education programmes are required to generate a desirable smoke-free environment for our next generation.

Assessment and control of children's ETS exposure should be carried out early, on a regular basis and in system level. The provision and referral of smoking cessation to the parents remain the key to protect children from exposure. In our study, it is alarming that very few (about 10%) of the participants had used local smoking cessation service and received cessation medications previously. Health care professionals are expected to play an important role in encouraging and referring smoking parents to cessation service. The underlying reasons for smokers' low adoption rate of conventional medical model to quit could be complex. Smokers might believe that quitting is their personal responsibility and they might perceive quitting unassisted to be the “right” or “better” choice (34). Half of the parents did not intend to quit in the next 6 months.

Smoke ban policy at home and in the private car was not widely adopted, the rates were much lower than the figures reported in other studies which were about 75% (11–13). Interestingly, about 80% of the smoking parents in this study reported favorable responses on other measures to reduce children's SHS exposure, for example, opening the door/window when smoking at home and keeping distance from the children when smoking. Nonetheless, favorable response for practices regarding children's THS exposure (those washing practice) was much lower (only 11% of the parents reported favorable responses on at least 70% of the items). Although 84.1 and 75.2% of the smoking parents exhibited good knowledge level of the concepts of SHS and THS, respectively, similar knowledge level did not result in concordant good practice. The lack of associations of knowledge and attitude with establishment of household smoke-free policy demonstrated that good parental knowledge and attitude may not promise a smoke free environment (19, 24). This may explain why some previous ETS reduction programmes failed to reduce children's exposure by parental education alone (35, 36). Most of these projects focused on advice on practice only, but did not pay much attention on exploring parents' ambivalence and barriers to translate knowledge and attitude to practice. Interventions helping to translate knowledge and attitude into better practice are warranted. The observed suboptimal parental practice on THS-related measures also showed areas for improvement. Additional effort would be needed to reduce children's THS exposure together with strong smoking cessation support and delivering of the information of remediation strategies to smoking parents appropriately in future practice.

Parents exhibited a low level of knowledge regarding the consequences from ETS exposure, but a relatively higher level of knowledge about tobacco use and the benefit of cessation. Moreover, parental knowledge on general hazards of tobacco exposure and specific hazards on children was especially insufficient. Relatively more parents knew that the tobacco exposure could be harmful to children's respiratory diseases, while fewer of them knew the hazards on other systems such as cardiovascular system, and even fewer of them knew the potential effects of ETS on children's cognitive ability. Parents generally demonstrated better knowledge in SHS than that in THS. Although more parents knew that they should not smoke in front of the children, they did not realize that their children could still be harmed by THS. The identified knowledge gaps should be highlighted in future parental tobacco control programme that more focus could be put on pediatric-specific health hazards from ETS exposure and those from THS exposure. Low parental knowledge about the available smoking cessation service and medications was also identified, which was consistent with the low utilization rate. Improved propaganda and doctor engagement are needed to promote utilization rate.

For parental attitudes, the exhibited parental fear toward nicotine withdrawal symptoms highlighted the need of pharmacological interventions, and the fear toward medications' side effects calls for appropriate counseling and support by the healthcare professionals. In addition, from the perspectives of many parents, information, and support from healthcare professionals are vital to encourage them to change. The barriers to behavioral change identified in the attitude section should be taken into consideration for future intervention development.

It is important to note that although the total practice scores and the THS-related practice scores were significantly associated with the private smoke-free policy, there was no relationship between SHS-related practice and home smoke-free policy likely because of our survey design. In our survey, parents who reported that smoking was banned in their home would automatically respond “not-applicable” for the SHS items (measures related to smoking inside the homes), and therefore explained the lack of associations between private smoke-free policy and SHS-related practice. On the other hand, parents with home smoke-free policy likely had more protective practice regarding children's ETS exposure, which might explain the association with protective practice regarding THS exposure.

The non-random sampling and the small sample size were the major limitations of this hospital-based study. Our participants were from a smoking reduction RCT that the recruitment was likely biased to those who were more motivated to change their smoking behaviors. Moreover, we did not have non-smoking control group. We performed convenience sampling by recruiting the parents of children who attended our pediatric service in the Prince of Wales Hospital, which is a major teaching hospital providing tertiary health care service to a representative population size in Hong Kong. However, the hospital-based setting limited the generalizability of the study findings to community population. The sample size was small and thus limited the statistical power. Furthermore, our KAP scale was self-developed. To advance the tool, a standardized international KAP scale should be developed. This can be achieved under the joint efforts of researchers and health care professionals from all over the world. Finally, the implications of unmeasured factors such as parental perceived norms or self-efficacy should be considered.

## Conclusions

The majority of the smoking parents were unwilling to quit in a short time. Smoke-free policy at home and in private car were not yet widely adopted. The parental KAP were generally poor among smoking parents in Hong Kong. Messages of recommended practice should be delivered to smoking parents appropriately. Due to the high addictiveness of nicotine, strong cessation support should also be provided. The identified smoking parents' perceived susceptibility and severity of children's tobacco exposure, benefits of and barriers to smoking cessation as well as the identified predictors for more favorable KAP should be taken into consideration when designing future ETS reduction interventions.

## Data Availability Statement

The datasets used in the current study are available from the corresponding author upon reasonable request and with permission of The Chinese University of Hong Kong. However, restrictions apply and the data are not publicly available.

## Ethics Statement

This study was conducted in accordance with the Declaration of Helsinki and was approved by the Joint Chinese University of Hong Kong-New Territories East Cluster Research Ethics Committee (CRE 2016.024-T). Written informed consent to participate in this study was provided by the smoking parents.

## Author Contributions

SD designed the study, collected data, carried out the data analyses, drafted the initial manuscript, reviewed and revised the manuscript. CA contributed to the study design and data analyses, interpreted the data and critically reviewed the manuscript for important intellectual content. MC and RK, contributed to the study design, supervised the biochemical analysis, interpreted the data and critically reviewed the manuscript for important intellectual content. AL contributed to the study design and subject recruitment, interpreted the data and critically reviewed the manuscript for important intellectual content. KC conceptualized and designed the study, recruited the subjects, supervised data collection and analyses, interpreted the data and critically reviewed the manuscript for important intellectual content. All authors approved the final manuscript as submitted and agreed to be accountable for all aspects of the work.

## Funding

This study was supported by the Research Fellowship Scheme, Health and Medical Research Fund (Grant Number: 01150077) from the Food and Health Bureau, Hong Kong SAR, China.

## Conflict of Interest

The authors declare that the research was conducted in the absence of any commercial or financial relationships that could be construed as a potential conflict of interest.

## Publisher's Note

All claims expressed in this article are solely those of the authors and do not necessarily represent those of their affiliated organizations, or those of the publisher, the editors and the reviewers. Any product that may be evaluated in this article, or claim that may be made by its manufacturer, is not guaranteed or endorsed by the publisher.
