# Supplementary material for: Parental Knowledge, Attitude, and Practice on Tobacco Use, Smoking Cessation, and Children's Environmental Tobacco Smoke Exposure
Source: Front Public Health. 2021 Nov 24;9:733667. doi: 10.3389/fpubh.2021.733667 (PMC8653904; doi:10.3389/fpubh.2021.733667)
Supplement: Supplementary file 1 [file Table_1.DOCX]

**Supplementary Table 1. Factors associated with parental KAP - univariate analyses (N=145)**

|  | Smoke policy at home  (yes vs. no) |  | Parental practice regarding children’s ETS exposure |  | Total Knowledge scores |  | Total Attitude  scores |  |
| --- | --- | --- | --- | --- | --- | --- | --- | --- |
| Parental characteristics | Odds ratio  (95% CI) | P value | Correlation coefficient r  (95% CI) | P value | Correlation coefficient r  (95% CI) | P value | Correlation coefficient r  (95% CI) | P value |
| Parental age (yrs.) | 0.97 (0.92, 1.02) | 0.18 | -0.18 (-0.31, -0.01) | **0.04** | -0.10 (-0.32, 0.08) | 0.25 | -0.14 (-0.39, 0.03) | 0.09 |
| Sex of smoking parent:  Female  Male | 1  1.75 (0.64, 4.81) | 0.28 | 0.03 (-2.31, 3.34) | 0.72 | 0.03 (-3.06, 4.48) | 0.71 | 0.008 (-3.80, 4.18) | 0.93 |
| Parental Education level:  Secondary school or below  Tertiary education or above | 1  1.17 (0.51, 2.68) | 0.71 | 0.06 (-1.51, 3.40) | 0.45 | 0.05 (-2.43, 4.26) | 0.59 | 0.03 (-2.96, 4.03) | 0.76 |
| Parental occupation:  Not employed  Employed | 1  1.74 (0.52, 5.86) | 0.37 | 0.16 (-0.08, 6.59) | 0.06 | 0.04 (-3.37, 5.58) | 0.63 | 0.02 (-4.14, 5.35) | 0.80 |
| Monthly household income > HKD20,000 | 1.38 (0.67, 2.81) | 0.38 | 0.18 (0.15, 4.29) | **0.04** | 0.12 (-0.87, 4.27) | 0.17 | 0.14 (-0.42, 5.46) | 0.09 |
| Parental current or previous chronic medical conditions | 0.52 (0.24, 1.12) | 0.10 | 0.03 (-0.78, 2.61) | 0.71 | -0.008 (-3.10, 2.81) | 0.93 | -0.01 (-3.32, 2.87) | 0.89 |
| Parental average daily smoking consumption:  1-10  11-20  21 or more | 1  0.72 (0.35, 1.48)  0.52 (0.14, 1.92) | 0.37  0.33 | -0.07 (-2.34, 0.97) | 0.42 | -0.08 (-3.30, 1.28) | 0.39 | -0.12 (-3.99, 0.67) | 0.16 |
| Parental nicotine dependence level (FTND score^&^) | 0.76 (0.64, 0.91) | **0.003** | -0.20 (-1.07, -0.10) | **0.02** | 0.02 (-0.60, 0.75) | 0.82 | -0.03 (-0.83, 0.58) | 0.73 |

**Supplementary Table 1. Factors associated with parental KAP - univariate analyses (N=145) (Cont.)**

|  | Smoke policy at home  (yes vs. no) |  | Parental practice regarding children’s ETS exposure |  | Total Knowledge scores |  | Total Attitude  scores |  |
| --- | --- | --- | --- | --- | --- | --- | --- | --- |
| Children’s characteristics | Odds ratio  (95% CI) | P value | Correlation coefficient r  (95% CI) | P value | Correlation coefficient r  (95% CI) | P value | Correlation coefficient r  (95% CI) | P value |
| Children’s age (years) | 0.97 (0.89, 1.05) | 0.43 | -0.25 (-0.60, -0.13) | **0.003** | -0.13 (-0.57, 0.07) | 0.13 | -0.12 (-0.56, 0.10) | 0.16 |
| Children’s sex:  Female  Male | 1  1.02 (0.52, 2.22) | 0.96 | 0.03 (-1.61, 2.43) | 0.69 | -0.04 (-3.37, 2.09) | 0.64 | 0.05 (-2.06, 3.66) | 0.58 |
| Existing chronic medical conditions^#^ | 0.54 (0.17, 1.70) | 0.29 | -0.04 (-4.29, 2.80) | 0.68 | -0.08 (-7.02, 2.57) | 0.36 | -0.10 (-3.23, 0.87) | 0.26 |
| Existing chronic respiratory tract diseases^^^ | 1.01 (0.47, 2.15) | 0.98 | -0.24 (-5.43, -0.92) | **0.006** | -0.17 (-6.00, 0.03) | **0.05** | -0.16 (-3.71, 0.17) | 0.07 |
| Existing chronic medical conditions except for RTI | 2.03 (0.83, 4.97) | 0.12 | 0.015 (-2.46, 2.92) | 0.87 | -0.007 (-3.75, 3.45) | 0.93 | -0.12 (-3.91, 0.71) | 0.17 |
| Current or previous allergic rhinitis | 0.95 (0.45, 2.22) | 0.95 | -0.14 (-4.24, 0.32) | 0.09 | -0.14 (-5.67, 0.48) | 0.10 | -0.14 (-5.94, 0.50) | 0.10 |
| Current or previous asthma | 0.35 (0.10, 1.31) | 0.12 | -0.10 (-5.24, 1.26) | 0.23 | -0.10 (-7.20, 1.73) | 0.23 | -0.13 (-0.82, 0.91) | 0.12 |
| Current or previous eczema | 0.78 (0.32, 1.90) | 0.59 | -0.05 (-3.35, 1.83) | 0.56 | -0.60 (-4.77, 2.24) | 0.48 | -0.08 (-5.33, 1.97) | 0.37 |
| Current or previous chronic lung disease | 0.97 (0.16, 6.00) | 0.97 | -0.04 (-6.67, 4.22) | 0.66 | -0.11 (-13.25, 2.82) | 0.20 | -0.18 (-15.72, -0.59) | **0.04** |
| Parental perception on child’s health status (Scale of 1-5) | 1.24 (0.86, 1.80) | 0.25 | 0.09 (-0.46, 1.65) | 0.27 | 0.005 (-1.41, 1.51) | 0.95 | 0.05 (-1.04, 1.98) | 0.54 |
| Need of long-term medication | 0.72 (0.27, 1.93) | 0.52 | -0.03 (-3.24, 2.36) | 0.76 | -0.06 (-4.94, 2.47) | 0.51 | -0.05 (-4.83, 2.76) | 0.59 |
| Premature at birth (<37 weeks’ gestation) | 1.73 (0.67, 4.47) | 0.26 | 0.004 (-2.63, 2.77) | 0.96 | -0.12 (-6.16, 1.00) | 0.16 | -0.14 (-6.89, 0.66) | 0.11 |
| Breastfed ever | 1.69 (0.81, 3.57) | 0.17 | 0.15 (-0.27, 4.27) | 0.08 | 0.30 (2.37, 8.25) | **<0.001** | 0.28 (2.18, 8.37) | **0.001** |
| Have other household smoker(s) besides the smoking parents | 0.61 (0.22, 1.70) | 0.35 | -0.02 (-3.29, 2.48) | 0.78 | -0.01 (-4.15, 3.57) | 0.88 | 0.009 (-3.86, 4.31) | 0.91 |

**Supplementary Table 2. Factors associated with parental KAP - multivariate analyses* (N=145)**

|  | Smoke policy at home  (yes vs. no)^@^ |  | Parental practice regarding children’s ETS exposure^^^ |  | Total Knowledge scores^&^ |  | Total Attitude  Scores# |  |
| --- | --- | --- | --- | --- | --- | --- | --- | --- |
| Parental characteristics | Adjusted Odds ratio  (95% CI) | P value | Adjusted Correlation coefficient r  (95% CI) | P value | Adjusted Correlation coefficient r  (95% CI) | P value | Adjusted  Correlation coefficient r (95% CI) | P value |
| Parental age (years) | 0.96 (0.87, 1.05) | 0.37 | 0.03 (-0.19, 0.25) | 0.79 | -0.12 (-0.44, 0.14) | 0.31 | -0.18 (-0.52, 0.07) | 0.13 |
| Sex of smoking parent:  Female  Male | 1  5.48 (0.47, 64.57) | 0.18 | -0.12 (-7.16, 2.97) | 0.41 | -0.08 (-7.80, 4.49) | 0.58 | -0.10 (-8.66, 4.15) | 0.49 |
| Parental Education level:  Secondary school or below  Tertiary education or above | 1  2.16 (0.64, 7.37) | **0.04** | -0.12 (-5.19, 1.30) | 0.24 | 0.01 (-4.07, 4.46) | 0.93 | 0.02 (-3.95, 4.86) | 0.84 |
| Parental occupation:  Not employed  Employed | 1  2.64 (0.30, 23.16) | 0.38 | 0.15 (-2.50, 8.99) | 0.27 | 0.03 (-6.63, 8.35) | 0.82 | 0.03 (-6.84, 8.64) | 0.82 |
| Monthly household income > HKD20,000 | 1.08 (0.36, 3.20) | 0.89 | 0.15 (-0.93, 4.85) | 0.18 | 0.20 (-0.46, 7.10) | 0.09 | 0.21 (-0.18, 7.61) | 0.06 |
| Parental nicotine dependence level (FTND score^#^) | 0.70 (0.53, 0.93) | **0.02** | -0.15 (-1.18, 0.30) | 0.24 | -0.03 (-0.92, 0.69) | 0.78 | -0.03 (-0.95, 0.71) | 0.77 |
| Children’s characteristics | Odds ratio  (95% CI) | P value | Correlation coefficient r  (95% CI) | P value | Correlation coefficient r  (95% CI) | P value | Correlation coefficient r  (95% CI) | P value |
| Children’s age (yrs.) | 0.95 (0.77, 4.72) | 0.45 | -0.11 (-0.52, 0.20) | 0.39 | -0.02 (-0.50, 0.44) | 0.90 | 0.03 (-0.43, 0.53) | 0.84 |
| Children’s sex:  Female  Male | 1  1.90 (0.77, 4.72) | 0.17 | -0.001 (-2.47, -2.44) | 0.99 | 0.09 (-1.70, 4.79) | 0.35 | 0.12 (-1.21, 5.44) | 0.21 |
| Existing chronic respiratory tract diseases^^^ | 0.56 (0.18, 1.72) | 0.30 | -017 (-5.33, 0.87) | 0.16 | -0.18 (-7.19, 0.94) | 0.13 | -0.16 (-7.21, 1.18) | 0.16 |
| Parental perception on child’s health status (Scale of 1-5) | 0.97 (0.57, 1.67) | 0.92 | 0.11 (-0.71, 2.16) | 0.32 | -0.09 (-2.75, 1.10) | 0.40 | -0.06 (-2.51, 1.38) | 0.57 |
| Premature at birth (<37 weeks’ gestation) | 2.00 (0.55, 7.30) | 0.29 | 0.004 (-3.33, 3.46) | 0.97 | -0.08 (-6.21, 2.69) | 0.43 | -0.10 (-7.01, 2.13) | 0.29 |
| Breastfed ever | 1.72 (0.69, 4.29) | 0.25 | 0.17 (-0.39, 4.81) | 0.10 | 0.29 (1.73, 8.60) | **0.004** | 0.29 (1.83, 8.85) | **0.003** |
| Have other household smoker(s) besides the smoking parents | 0.42 (0.10, 1.75) | 0.23 | 0.07 (-2.45, 4.93) | -0.51 | -0.07 (-6.37, 3.27) | 0.53 | -0.05 (-6.16, 3.79) | 0.64 |

*4 regression models were built: the dependent variable is “smoke policy at home”, or “Parental practice regarding children’s ETS exposure”, or “Total knowledge scores” or “Total attitude scores”, while independent factors for each regression model including those parental characteristics and children’s characteristics.

^@^The logistic regression model’s Nagelkerke R square=0.27; ^^^The linear regression’s R square=0.18; ^&^The linear regression’s R square=0.18; ^#^The linear regression’s R square=0.20
